# Sellar brain tumour co-existing with a left posterior communicating aneurysm causing ptosis: lessons learnt (case report)

**DOI:** 10.11604/pamj.2023.44.60.38596

**Published:** 2023-01-31

**Authors:** Chiazor Onyia, Omotayo Ojo, Bruno Arekhandia

**Affiliations:** 1 Department of Surgery, Lagoon Hospitals, Lagos, Nigeria,; 2 Neurosurgery Unit, Department of Surgery, Lagos University Teaching Hospital, Lagos, Nigeria

**Keywords:** Posterior communicating artery aneurysm, sellar brain tumour, multiple brain lesions, aneurysm, case report

## Abstract

Posterior communicating aneurysm (PCOM) commonly presents with ptosis of the eye. This is so also with space occupying lesion compressing the oculomotor nerve. It is quite uncommon for a patient to have both lesions at the same anatomical space concurrently causing ptosis. If undiagnosed before surgical intervention, surgical complications from such a complex neurosurgical problem can be extremely devastating. We share our recent experience with confusing clinical presentation and subsequent treatment of a sellar brain mass co-existing with a left posterior communicating artery aneurysm at the same location in a 48 years old woman who was successfully managed with left pterional craniotomy and clipping of the PCOM aneurysm followed by gross total excision of the lesion. She improved with full resolution of left ptosis within 2 months. The histology revealed WHO Grade 1 psammomatous meningioma. Although similar sorts of pathologies occurring together have been described in the literature, this unique experience underscores the importance of thoroughly evaluating neurosurgical cases clinically irrespective of findings from radiologic investigations in order to prevent unexpected intra-operative disasters and embarrassment. Moreso, this is important particularly in Africa where advanced investigations to easily identify such complex pathologies are not readily available.

## Introduction

PCOM aneurysm is well known to be a common cause of ptosis. This clinical manifestation is also classical with any space occupying lesion compressing the oculomotor nerve. It is quite uncommon for a patient to have combination of both lesions at the same time causing ptosis. Although quite rare, neurovascular lesions could co-exist with brain tumours such as meningioma (29.3-44%), glioma (27.5-38%), pituitary adenoma (11-20.6%), lymphoma, craniopharyngioma, chordoma, epidermoid tumor, dermoid tumour, as well as choroid plexus adenoma [1]. If undiagnosed before surgical intervention, surgical complications from such complex neurosurgical problems can be extremely devastating including death due to uncontrollable bleeding from the aneurysm [2]. However, risk for such unwanted situations appears to be on the increase in recent times due to an increasing over-dependence on imaging among other investigative modalities and less attention to clinical details in arriving at a diagnosis. Although intracranial pathologies occurring together have been well documented in the literature [1-7], we share a unique experience and lessons from the case of a sellar brain mass co-existing with a left PCOM aneurysm at the same location in a 48 years old woman to emphasize the importance of clinical evaluation despite radiologic findings on investigation.

## Patient and observation

**Patient information**: a 48-year-old woman was referred to the facility for a transsphenoidal sellar tumor removal. She was a known hypertensive patient who presented on account of sudden onset of headache with complete left ptosis and diplopia. Her medical, family and other relevant history were not remarkable.

**Clinical findings**: her vital signs were within limits. Clinical examination revealed left oculomotor nerve palsy with ophthalmoplegia. Otherwise, she was fully conscious with no other cranial nerve deficits. Motor and sensory examination revealed no remarkable findings.

**Figure 1:**
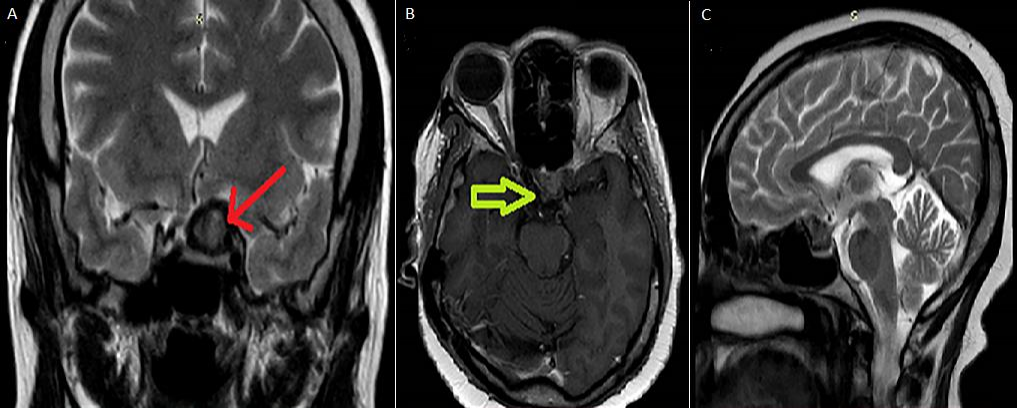
A,B,C) T2 coronal view, axial view of T1 contrast and T2 sagittal view respectively of the patient´s brain MRI scan demonstrating a sellar space-occupying lesion with moderate contrast enhancement (green block arrow in B) and a hypointense periphery (red line arrow in A)

**Figure 2:**
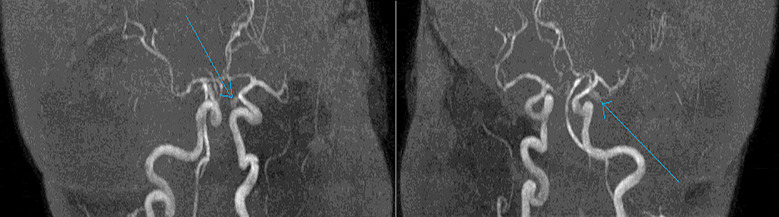
rotated sagittal views of arterial phase on CT angiogram demonstrating the left posterior communicating artery aneurysm (blue line arrow)

**Diagnostic assessment**: brain magnetic resonance imaging (MRI) done prior to presentation showed a sellar space-occupying lesion with homogenous contrast enhancement and a hypointense periphery (Figure 1 A,B,C). However, her clinical presentation as well as the imaging findings raised the suspicion of possibility of a vascular pathology as the underlying cause of her symptoms. Investigation with a 3-dementional computed tomography (CT) angiogram revealed an adjacent saccular left posterior communicating aneurysm (Figure 2 A,B). Endocrine tests confirmed normal pituitary function.

**Figure 3:**
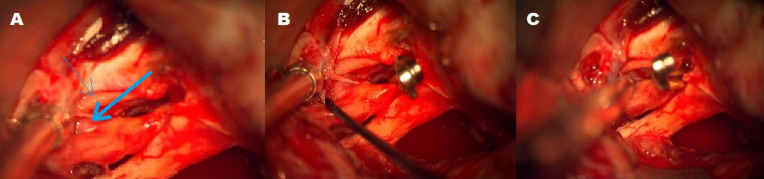
at surgery; aneurysm immediately before and after clip application; A) oculomotor nerve (thin arrow); left PCOM aneurysm (thick arrow); B) application of temporal clip on left ICA for vascular control; C) application of permanent clip across the neck of left PCOM aneurysm

**Therapeutic interventions**: she was offered left pterional craniotomy and clipping of the aneurysm (Figure 3 A,B,C) followed by gross total excision of the lesion at the same surgery.

**Follow-up and outcome**: the histology revealed WHO Grade 1 psammamatous meningioma. Her ptosis completely resolved and she made full recovery.

**Informed consent**: the patient consented to the procedure.

## Discussion

This is a clear illustration of the importance of thoroughly evaluating available neuroimaging for each neurosurgical case with the clinical presentation for that case in perspective. In present-day medical practice, there appears to be an increasing over-dependence on imaging among other investigative modalities and less attention to clinical details in arriving at a diagnosis. Although her brain MRI scan revealed a sellar mass, the suddenness of her symptoms is not typically characteristic of brain tumours. Symptoms from brain neoplasm are generally known to be gradually or rapidly progressive depending on the level of aggressiveness while vascular problems are commonly sudden in clinical manifestation. Second, homogenous contrast enhancement and a hypointense periphery (Figure 1 A) are not pathognomonic of only meningioma. A few other lesions (particularly vascular ones) could have similar features. Third, advanced investigation modalities which can easily identify and separately distinguish such complex pathologies from each other are significantly lacking in many low-and-middle-income- countries (LMICs) especially Africa.

Of the brain tumours associated with cerebral aneurysms in neurosurgical practice, meningiomas are the commonest [6]. Several hypotheses have been proposed to explain the possible causal association of tumour with aneurysm, including increase in the directional blood flow due to a higher blood supply (meningioma, malignant glioma), hormonal influence of IGF-1 on artery walls (artery dilation, atherosclerotic and degenerative changes) and genetic predisposition, tumour invasion and tumour-directed neo-vascularization, and previous intracranial tumour surgery causing traumatic aneurysms [1,3,6]. An aneurysm may develop on the artery feeding a large meningioma due to the haemodynamic stresses on these feeding arteries caused by the increased blood flow to the meningioma [6]. In the case we reported, the meningioma was however relatively small. Several strategic options exist for management according to the symptoms produced by the lesion, the locations of tumour and aneurysm, as well as the nature of the neoplasm itself [5]. For patients with concurrent brain tumour, simultaneous aneurysm clipping is technically more challenging and riskier while on the other hand, preoperative coiling with subsequent tumour surgery has been demonstrated to be relatively safer and more efficient [7]. To avoid the rupture of aneurysmal sac during the removal of the meningioma, if the aneurysm is near or adjacent to the meningioma, clipping of aneurysm should be performed first before the removal of meningioma [4].

## Conclusion

Various intracranial brain pathologies occurring together have been described in the literature [1-7]. However, this is a unique case in view of the points highlighted. In situations of suspected multiple pathologies at the same intracranial location, it is important to thoroughly evaluate all imaging in tandem with the clinical presentation for accurate diagnosis in order to ensure that it is safely treated without unexpected intra-operative disasters or complications as much as possible. This is particularly helpful in Africa and other low-and-medium-income settings where advanced investigations which could easily identify such complex pathologies are not readily available.
